# Low-Velocity Impact Response of Sandwich Structure with Triply Periodic Minimal Surface Cores

**DOI:** 10.3390/polym17060712

**Published:** 2025-03-07

**Authors:** Dong Wei, Shaoan Li, Laiyu Liang, Longfei Sun, Yaozhong Wu

**Affiliations:** 1 Alphapec Instrument (Hubei) Co., Ltd., Yichang 443000, China; wdhust2011@sina.com; 2 MOTUSTECHS (WuHan) Co., Ltd., Wuhan 430073, China; 3 Wuhan Second Ship Design and Research Institute, Wuhan 430205, China; 4 China Ship Development and Design Center, Wuhan 430064, China; 5 School of Automobile and Traffic Engineering, Wuhan University of Science and Technology, Wuhan 430081, China

**Keywords:** low-velocity impact, triply periodic minimal surfaces, sandwich structures, finite element simulation

## Abstract

Triply periodic minimal surface (TPMS) sandwich structures were proposed based on the TPMSs. The test samples for the TPMS sandwich were prepared using Multi Jet Fusion (MJF) with PA12 as the base material. Their low-velocity impact responses were investigated using experimental tests and numerical simulation. The effect of structural parameters (relative density, panel thickness, impact energy, and TPMS core) on the impact performance of the sandwich structures was analyzed through parameter studies. The results indicate that the peak load and stiffness of the sandwich structure increase with the increase in relative density, panel thickness, and impact energy. Among three types of TPMS core sandwich structures, the Diamond sandwich structure exhibits the biggest peak load and best impact resistance.

## 1. Introduction

A sandwich structure is a class of composite structures that exhibit excellent specific bending stiffness and strength, energy-absorption capacity, and flexibility for design [1,2,3]. These structures are widely used in aerospace [4], automotive [5,6], and marine engineering [7,8]. The sandwich structures are vulnerable to various impact events, such as bird strikes, dropped tools, and floating ice [9]. Severe damage induced by these impact events can significantly reduce their load-bearing capacity, which may lead to failure of the whole structure. For this reason, the dynamic response of the sandwich structures under impact loading has drawn growing attention [10,11].

A sandwich structure consists of a thick but soft core and two thin but stiff panels. The core topologies and their base materials have a significant effect on the impact response of the sandwich structures [12]. Various cores have been used in the sandwich structures to enhance their impact resistance under impact loads, such as corrugated [13], honeycomb [14], and lattice cores [15,16]. For example, Huo et al. [17] proposed a foam-core sandwich structure and investigated its low-velocity impact performance using experimental tests and numerical simulations. The results found that the multi-layered sandwich had better crush force efficiency. Korupolu et al. [18] presented a new sandwich structure with hierarchical second-order hexagon honeycomb cores and investigated their impact performances using numerical simulations. It is found that the deflection of the back panels of the proposed hierarchical second-order hexagon honeycomb sandwich is smaller compared with the regular honeycomb sandwich. Parameter studies were conducted to derive the optimal structural parameters. Zhang et al. [19] studied the dynamic response of the tube-reinforced honeycomb sandwich using drop-weight impact tests and numerical simulations. They found that the metallic tube filler in the core of the sandwich can improve its stiffness and peak load. Yu et al. [20] studied the impact performance of corrugated sandwich structures using experimental, numerical, and theoretical methods. Three initial failure modes, face-sheet yield, face-sheet buckling, and core buckling were found, and failure maps were obtained using the theoretical method. Pan et al. [21] investigated the dynamic response of the thermoplastic composite corrugated sandwich panels using experimental tests and finite element (FE) analysis. The results indicated that the intersected corrugated sandwich panels exhibit better impact resistance compared to their counterparts. Jhou et al. [22] proposed a lattice-core sandwich structure and prepared the test samples using the 3D-printed method. The impact response was investigated by experimental tests. It was found that the structural parameters of the lattice core have a key effect on the dynamic responses of the sandwich structures. Taghipoor et al. [23] investigated the force–displacement relationship of the lattice-core sandwich panels using the 3D-printed samples. The findings indicate that the proper selection of core topology for the sandwich can improve its energy efficiency. Sukia et al. [24] proposed a new sandwich using a bio-inspired core and manufactured the test samples using the 3D-printing method. The low-velocity impact performance of sandwiches was investigated, and the results show that the proposed sandwich exhibits excellent impact resistance. Wen et al. [25] proposed a bamboo bio-inspired core sandwich and investigated their dynamic responses under low-velocity impact loading using the samples manufactured using the Fused Deposition Modeling method. It was found that the bamboo sandwich absorbed the most energy compared to its counterparts. Ejeh et al. [26] studied the dynamic response of the 3D-printed sandwich panels. The asymmetric sandwich with a thick back face sheet and functionally graded weak-to-strong core had the highest impact energy dissipation.

Recently, much attention has been drawn by researchers to the triply periodic minimal surfaces found in nature [27,28]. Yin et al. [29] found that TPMS sheet-based cellular structures exhibit higher energy-absorption capacity and mechanical properties. Zhu et. al. [30] proposed a diamond lattice of cylindrical shells using a mapping method and studied their energy-absorption performance. Zhang et al. [31] compared the mechanical and energy-absorption properties between the TPMS and lattice structures. The results show that the TPMS structures are better than the lattice structures in terms of mechanical and energy-absorption performances.

To further improve its mechanical energy-absorption properties for the sandwich structures, the TPMS cores were introduced to the sandwich design. Peng et al. [32] investigated the bending properties, failure mechanism, and energy-absorption performances of the TPMS core sandwich under three-point bending loading. They found that the sandwich structure with the Neovius core has better performance compared to other sandwich structures. Li et al. [33] prepared the sandwich panel with the TPMS core using Fused Deposition Modelling with composited reinforced with continuous fibers. Three-point bending experiments were conducted and FE models were developed for the proposed sandwich structures to study the mechanical properties. It was found the Schwarz Primary core sandwich structure has the best mechanical properties. Guo et al. [34] investigated the three-bending performances of the TPMS sandwich using experiments, theoretical analysis, and FE simulations. The results indicate that the TPMS sandwich has the potential to be applied in engineering applications. Fashanu et al. [35] studied the mechanical properties of the sandwich structure, which was constructed using two carbon-fiber-reinforced polymer (CFRP) face sheets and TPMS core. Wu et al. [36] studied the sound transmission loss properties of TPMS sandwiches using theoretical, numerical, and experimental methods. The results show that the Diamond TPMS core sandwich has the best acoustic performance. Lin et al. [37] investigated the sound insulation performances of the TPMS sandwich. They found that the TPMS sandwich structures exhibit good sound suppression capacities.

The mechanical, acoustic, and energy-absorption properties of the sandwich structures with TPMS core have been studied. However, the dynamic response of the sandwich structures with TPMS core under low-velocity loading has not been reported to date.

Therefore, the TPMS sandwich structure test samples were prepared using the 3D-print method, and their dynamic responses were investigated using drop-weight impact tests. The FE model for the low-velocity impact response of the TPMS sandwich structure is established and the accuracy of the model is verified using experimental results. The effect of structural parameters (relative density, panel thickness, impact energy, and TPMS core) on the impact performance of the TPMS sandwich structure was studied by parameter studies.

## 2. Materials and Methods

### 2.1. TPMS Sandwich Structure Design

Three TPMS surfaces, namely Primitive (P), Gyroid (G), and Diamond (D), are studied in this study, and these TPMS surfaces can be described by level set function as follows [2,27]:

Primitive:

(1) $$\phi_{P}\equiv \cos\left(X\right)+\cos\left(Y\right)+\cos\left(Z\right)=c$$

Gyroid:

(2) $$\phi_{G}\equiv \sin\left(X\right)\cos\left(Y\right)+\sin\left(Y\right)\cos\left(Z\right)+\sin\left(Z\right)\cos\left(X\right)=c$$

Diamond:

(3) $$\phi_{D}\equiv \cos\left(X\right)\cos\left(Y\right)\cos\left(Z\right)-\sin\left(X\right)\sin\left(Y\right)\sin\left(Z\right)=c$$

where $\phi_{P}$, $\phi_{G}$, and $\phi_{D}$ are the level set functions for the Primitive, Gyroid, and Diamond surfaces, respectively. X=x·2π/L, Y=y·2π/L, and Z=z·2π/L, x, y, z are spatial coordinates; L is the unit cell length of the TPMS surfaces; and c is a constant.

The sheet-network lattices were constructed by two minimal surfaces with opposite values ($\phi_{P,G,D}=\pm c$). The relative density (RD) of the lattices is calculated by dividing the volume of lattices ($V_{P,G,D}$) by the volume of the cube ($V_{\mathrm{cube}}$) occupied by the lattices.

**Figure 1:**
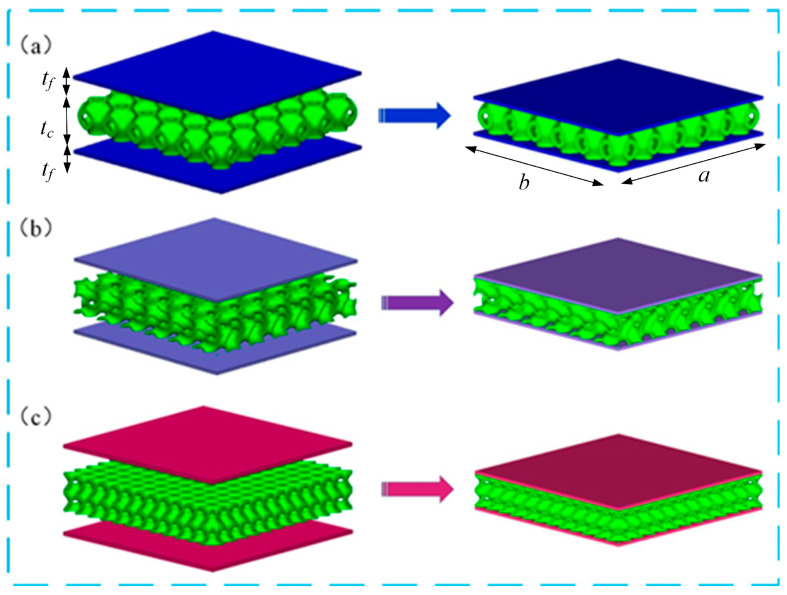
Construction of the TPMS sandwich structures: (**a**) P-TPMS sandwich, (**b**) G-TPMS sandwich, (**c**) D-TPMS sandwich.

The proposed TPMS sandwich structures consist of two panels and the TPMS cores, as shown in Figure 1. The TPMS core was built by arranging the unit cell of sheet-network lattices in three orientations as required. The length of the TPMS sandwich is a, and the width of the TPMS sandwich is b; the thickness of the two panels is $t_{f}$, and the thickness of the TPMS core is $t_{c}$.

### 2.2. Sample Preparation and Low-Velocity Impact Tests

Experimental samples were designed to explore the impact performances of the TPMS sandwich structures, and all the samples have the same geometric parameters: a=96 mm, b=96 mm, $t_{c}=16\;\mathrm{mm}$, and L=16 mm. Nine types of specimens were designed to study the effect of different geometric parameters and impact energy on the impact responses of sandwich structures, and the details of the samples are listed in Table 1. The test samples are named X-XX-XX-XX. Taking D-0.25-1.5-10 as an example, this name means that this is a D-TPMS core sandwich with RD=0.25, $t_{f}=1.5\;\mathrm{mm}$ and impact energy (E) of 10 J.

All the samples were prepared using Multi Jet Fusion (MJF) with base material of HP 3D high reusable material PA12 by Guangzhou 3DPlink Digital Technology Co., Ltd., (Guangzhou, China). The printed process parameters are listed in Table 2.

**Figure 2:**
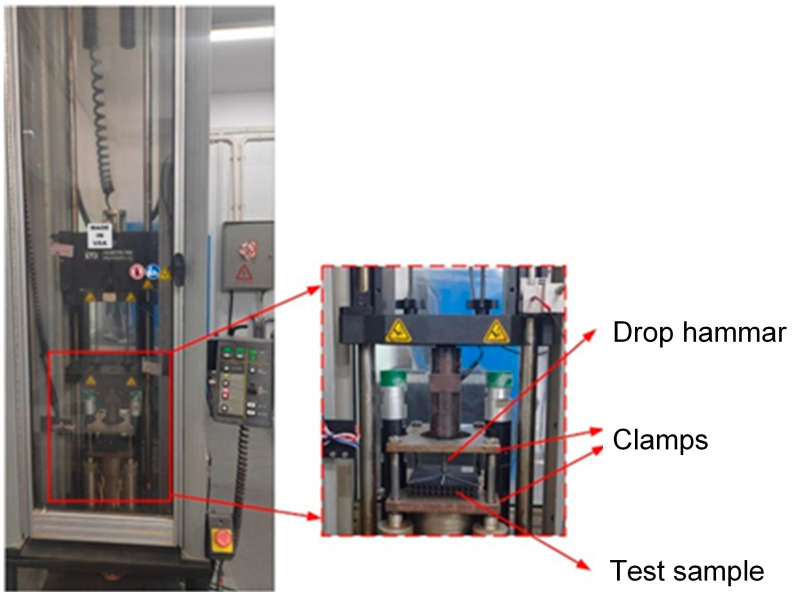
Low-velocity impact test setup.

According to the ASTM D7766/D7766M-11 standard issued by the American Society for Experiments of Materials [38], the drop-weight impact test was carried out on TPMS sandwich samples using the Instron Dynatup 9250HV (Boston, MA, USA) drop-weight impact testing machine, as shown in Figure 2. The experimental machine is mainly composed of a drop hammer system, a data-acquisition system, an anti-secondary impact system, and an operation platform.

In the low-velocity impact test, the mass of the drop hammer impact system is 17.55 kg. The specimen is fixed by moving up and down two square steel plate clamps with holes, and the drop hammerhead is a hemispherical punch with a diameter of 10 mm. The impact energy can be adjusted by changing the height of the punch to carry out low-velocity impact tests with different impact energies.

The data-acquisition system is used to carry out low-velocity impact experiments on sandwich structures in the experimental process. The force–time curve of the impact process is analyzed. In addition, the specimen after impact is cut along the half section by a cutting machine to observe the internal damage of the specimen.

### 2.3. Finite Element Simulation

**Figure 3:**
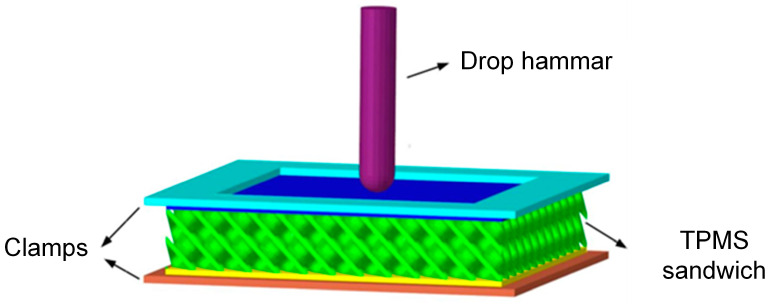
FE model of impact for the TPMS sandwich.

**Figure 4:**
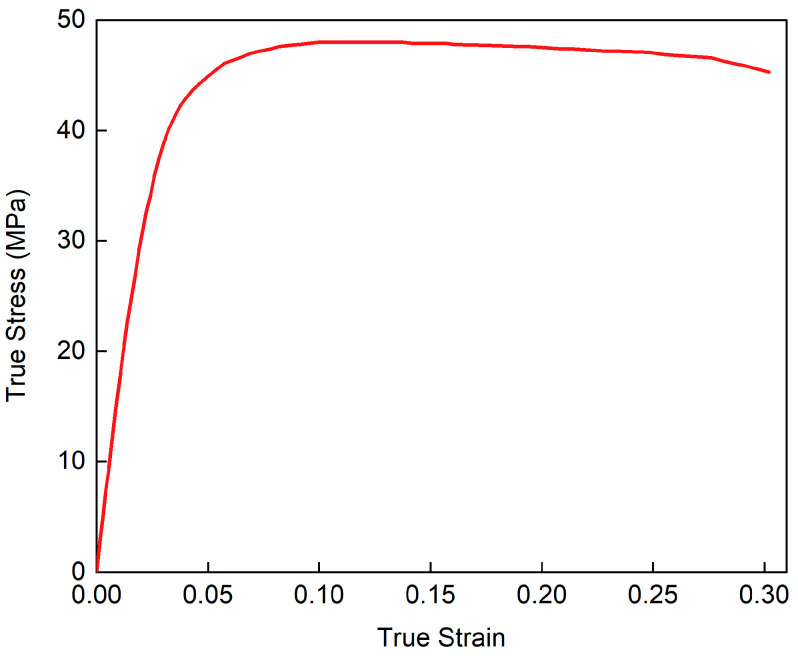
True stress–strain curves of 3D-printed PA12 material.

The finite element model is established using explicit nonlinear dynamics software LS-DYNA (Version 971R4) to simulate the response of the sandwich structure under low-velocity impact loading, as shown in Figure 3. The TPMS sandwich structure, punch, and upper and lower clamps are all modeled by solid elements. The punch and upper and lower clamps are modeled by the rigid material model (*MAT_20). The elastic-perfectly plastic material model (*MAT_03) is used to simulate the TPMS sandwich structure. The mechanical properties of PA12 are listed in Table 3. The tensile stress–strain curves of 3D-printed PA12 samples are presented in Figure 4. The failure model for the base material is defined based on the maximum principal strain failure criterion, and the failure strain is set to 0.3.

**Figure 5:**
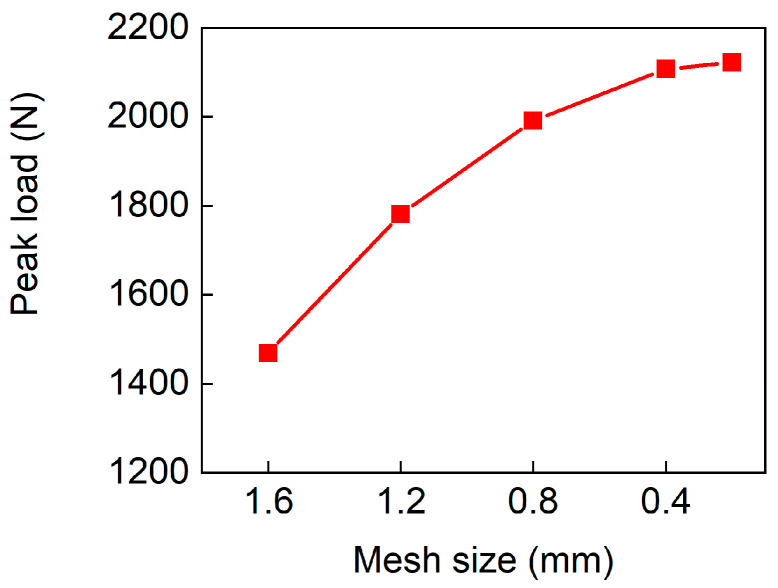
Convergence of the peak loads under different element sizes.

The convergence tests were conducted to obtain the optimal mesh size for the FE model. Taking the D-0.25-1.5-10 sample as the example, the convergence of the peak loads with different mesh sizes is presented in Figure 5. It can be seen that the difference between 0.4 mm and 0.3 mm is small. To reduce the computational time, a mesh size of 0.4 mm is adopted in this study.

An automatic face-to-face contact algorithm is adopted for the contact between the TPMS sandwich structure, punch, and clamp, and the self-contact of the TPMS sandwich structure itself is simulated using the automatic single-side contact algorithm. The dynamic and static friction coefficient for all contact are set to 0.3 [39,40].

Fixed constraints are imposed on the upper and lower clamps, the punch is only allowed to move in the vertical direction, and other degrees of freedom are fixed. The corresponding initial speed is set for the punch, which is consistent with the experimental tests.

## 3. Results

### 3.1. Experimental Results and Validation of the FE Model

**Figure 6:**
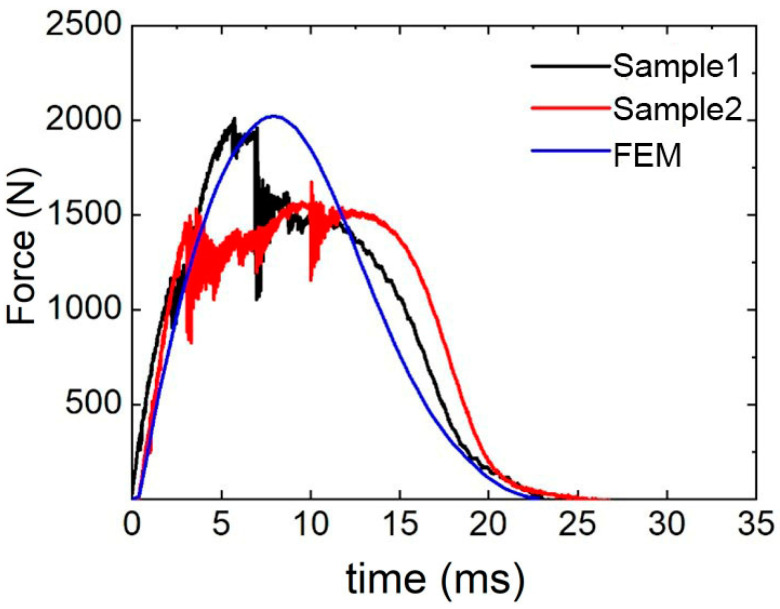
Comparison of force–time curves between test and simulation.

To verify the accuracy of the finite element model under low-velocity impact, The D-0.25-1.5-10 sandwich structure is taken as an example. Figure 6 presents the comparison of the test and simulation results for the D-0.25-1.5-10 sandwich structure.

The impact process mainly has three stages. In the first stage, the impact force increases linearly, and the impact energy is mainly absorbed by the deformation of the upper panel. With the increase in impact time, the impact force gradually reaches the maximum value. After reaching the ultimate strength of the upper panel, the upper panel begins to damage, resulting in a sudden drop in impact force.

In the second stage, after the drop in impact force, the upper panel loses its ability to bear the load because of the damage. However, with the energy absorbed by the core layer, a platform stage appears for the impact force.

In the third stage, the punch begins to rebound, and the impact force gradually decreases to zero.

The peak force obtained by finite element simulation is 2020.66 N, the average peak force obtained by experiment is 1841.73 N, and the relative error of peak load is 9.7%. The test and simulation force–time curves exhibit similar trends, and the results are in good agreement with each other. This shows that the simulation model has a certain accuracy and can accurately simulate the impact process.

### 3.2. Parameter Study

#### 3.2.1. Influence of Relative Density on Impact Performance of Sandwich Structure

**Figure 7:**
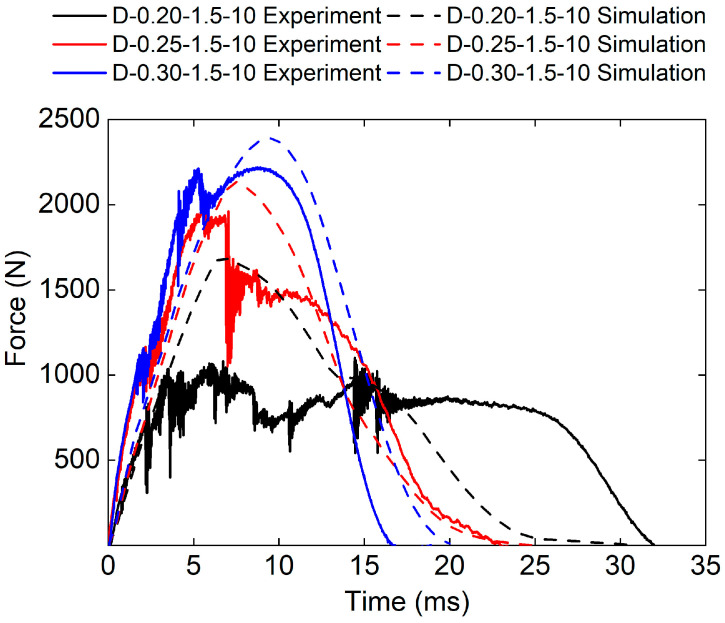
Experimental and simulation force–time curves of TPMS sandwich with different RD.

Figure 7 presents the experimental and numerical force–time curves of sandwich structures with different relative densities. It can be found that the force–time curves of the three sandwich structures have similar trends. The force–time curves obtained by FE analysis are in good agreement with the experimental results. Specifically, the experimental peak load of D-0.20-1.5-10 is smaller than the numerical peak load. This is because the relative density of the core is small and the core structure is prone to damage. It also can be found that the impact stiffness increased with the increase in the relative density of the core. The D-0.30-1.5-10 sandwich structure has the highest impact stiffness.

The experimental peak load of the D-0.30-1.5-10 sandwich structure is 2220.33 N. The experimental peak load of the D-0.25-1.5-10 sandwich structure is 2010.4 N. The experimental peak load of the D-0.20-1.5-10 sandwich structure is 1101.51 N. The simulated peak loads of the D-0.30-1.5-10, the D-0.25-1.5-10, and the D-0.20-1.5-10 sandwich structures are 2388.98 N, 2020.66 N, and 1355.59 N respectively. The relative errors between experimental peak loads and simulated peak loads are 7.6%, 0.5%, and 23.1%, respectively.

**Figure 8:**
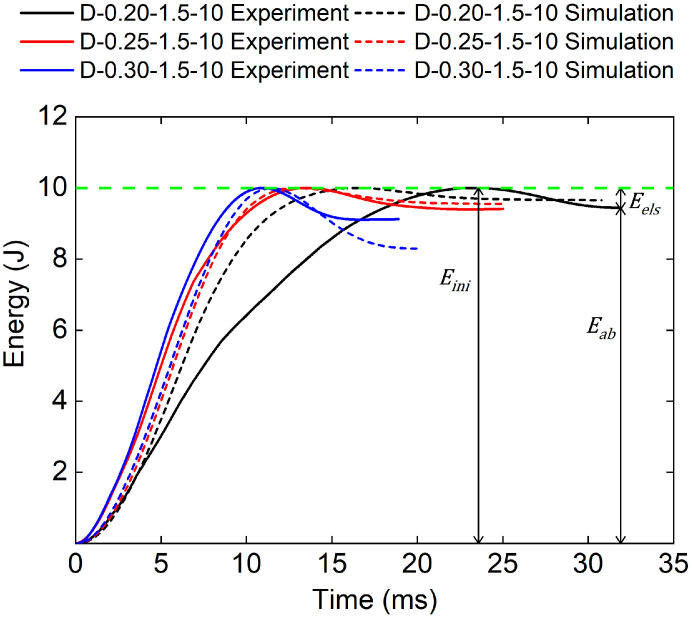
Comparison of energy–time curves between test and simulation of TPMS sandwich with different RD.

Figure 8 and Table 4 show the experimental and numerical results of TPMS sandwich structures with different relative densities. It can be seen that the initial kinetic energy ($E_{ini}$), consists of the absorbed energy ($E_{ab}$), and the elastic energy ($E_{els}$), was reached when the punch’s velocity was zero. Then, the punch begins to rebound to release the elastic energy ($E_{els}$). It can be seen that the whole impact time and $E_{ab}$ decreases with the increase in the relative density of the core. The reason may be that the bigger the relative density of the core, the stiffer the core structure, which leads to more elastic energy stored in the core structure.

**Figure 9:**
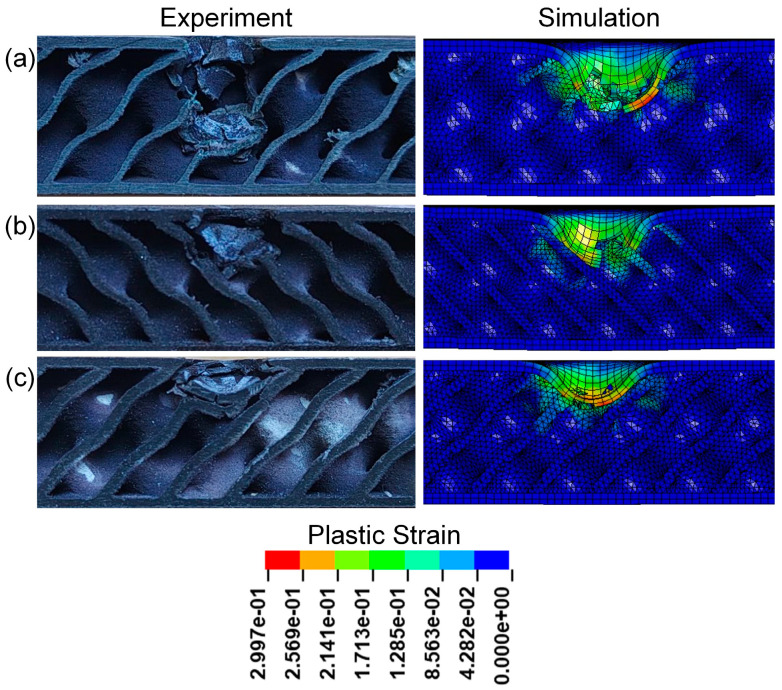
Comparison of the experimental and simulation impact damage of TPMS sandwich with different RD: (**a**) D-0.2-1.5-10; (**b**) D-0.25-1.5-10; (**c**) D-0.3-1.5-10.

Figure 9 presents the cross-sectional view of sandwich structures with different relative densities. It can be seen that obvious pits appear in the impact areas of TPMS sandwich structures after impact, mainly concentrated in the punch impact positions of TPMS sandwich structures. Among them, the D-0.20-1.5-10 sandwich structure has the most serious damage on the panel and core, and the D-0.30-1.5-10 sandwich structure has slight damage on the panel and core. However, the damage process is not well simulated for the D-0.20-1.5-10 sandwich, as shown in Figure 9a.

**Figure 10:**
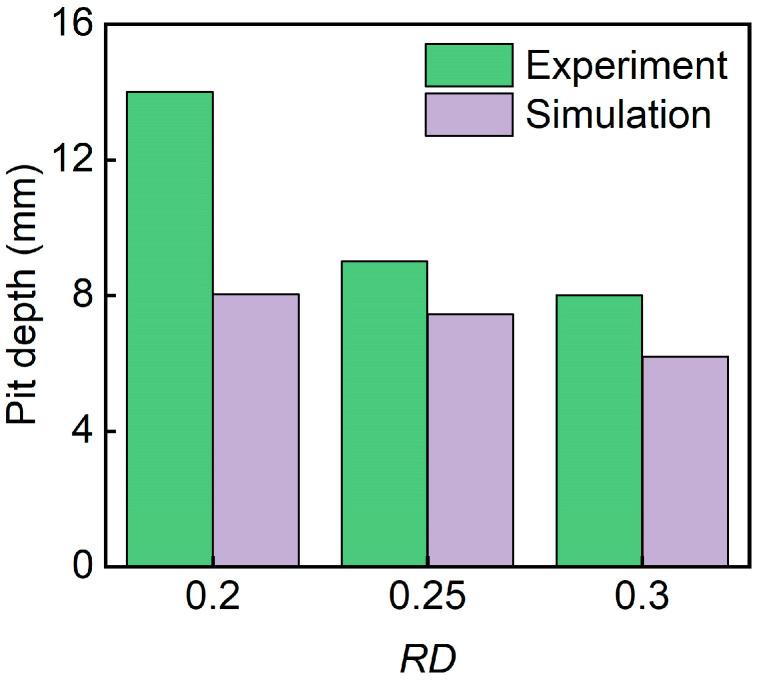
Comparison of the experimental and simulation pit depth of TPMS sandwich with different RD.

Figure 10 presents the pit depth of sandwich structures with different relative densities. It can be seen that pit depth decreases with the increase in the relative density of the core. The D-0.20-1.5-10 sandwich has the biggest pit depth.

#### 3.2.2. Influence of Panel Thickness on Impact Performance of Sandwich Structure

**Figure 11:**
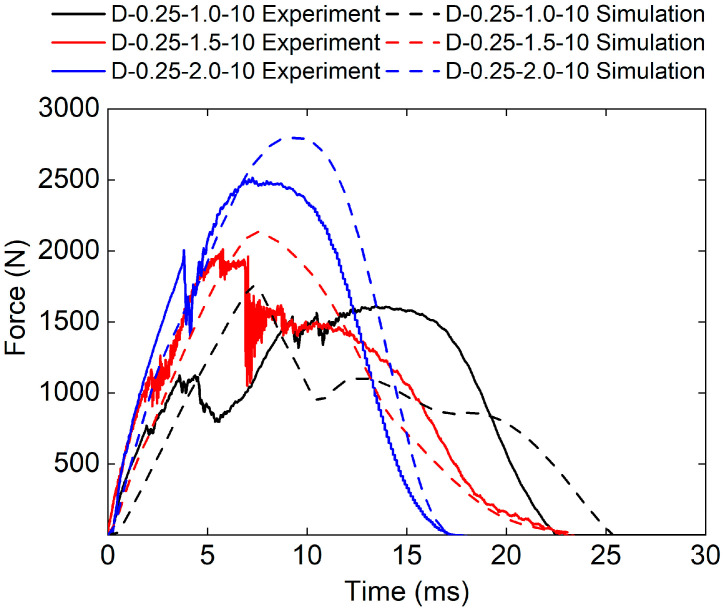
Experimental and simulation force–time curves of TPMS sandwich with different $t_{f}$.

Figure 11 presents the force–time curve for three sandwich structures with different panel thicknesses obtained from both the experimental and numerical methods. The force–time curves obtained by FE analysis are in good agreement with the experimental results. The results show that the impact stiffness increased with the increase in panel thickness. The D-0.25-2.0-10 sandwich structure has the highest impact stiffness.

The experimental peak loads of the D-0.25-1.0-10, D-0.25-1.5-10, and D-0.25-2.0-10 sandwich structures are 1612.26 N, 2010.4 N, and 2515.95 N, respectively. The simulated peak loads of the D-0.25-1.0-10, D-0.25-1.5-10, and D-0.25-2.0-10 sandwich structures are 1758.23 N, 2020.66 N, and 2797.36 N, respectively. The relative errors between experimental peak loads and simulated peak loads are 7.6%, 0.5%, and 23.1%, respectively.

**Figure 12:**
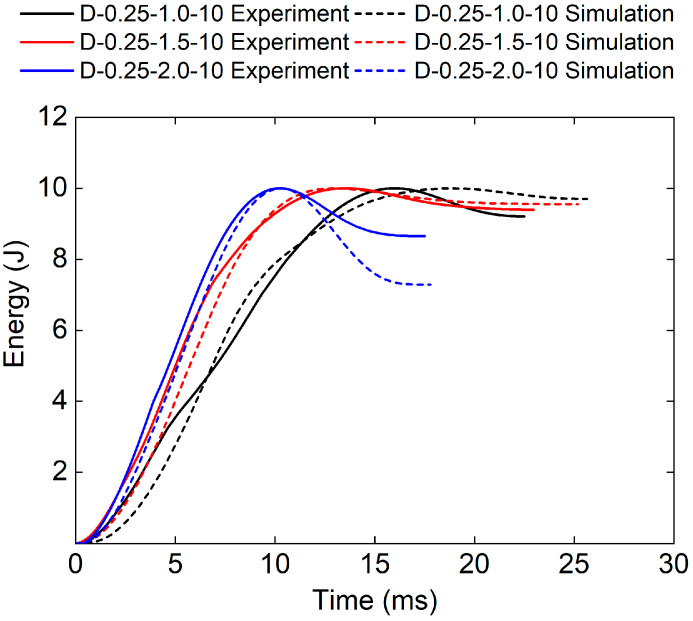
Comparison of energy–time curves between test and simulation of TPMS sandwich with different $t_{f}$.

Figure 12 and Table 5 show the experimental and numerical results of TPMS sandwich with different panel thicknesses. The whole impact time and $E_{ab}$ decreases with the increase in the panel thicknesses. The reason for this may be that the upper panel thickness is bigger which leads to higher-impact stiffness and more elastic energy stored in the upper panel.

**Figure 13:**
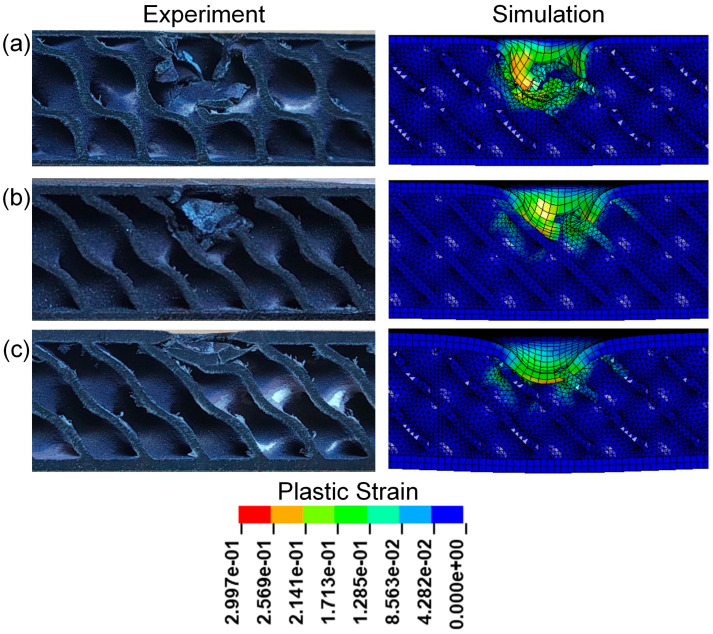
Comparison of the experimental and simulation impact damage of TPMS sandwich with different $t_{f}$: (**a**) D-0.25-1.0-10; (**b**) D-0.25-1.5-10; (**c**) D-0.25-2.0-10.

Figure 13 presents the cross-sectional view of sandwich structures with different panel thicknesses after impact. All the upper panels of sandwich structures were destroyed, and obvious pits appeared in the impacted areas of TPMS sandwich structures. Among them, the D-0.25-1.0-10 sandwich structure has the most serious damage to the panel and core, as shown in Figure 13a. As can be seen from Figure 13a,c, both the panel and core layers are fractured, and the D-0.25-1.5-10 sandwich structure is slightly more seriously damaged than the D-0.25-2.0-10 sandwich structure.

**Figure 14:**
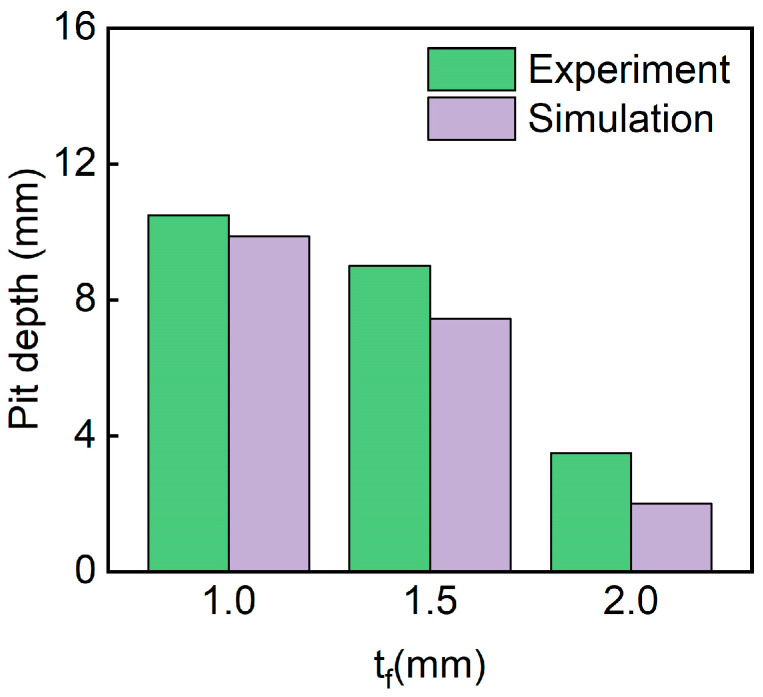
Comparison of the experimental and simulation pit depth of TPMS sandwich with different $t_{f}$.

Figure 14 shows the pit depth of sandwich structures with different panel thicknesses. It can be seen that pit depth decreased with the increase in panel thicknesses. The D-0.25-1.0-10 sandwich has the biggest pit depth. The simulated pit depths are in good agreement with the experimental pit depths.

#### 3.2.3. Influence of Impact Energy on Impact Performance of Sandwich Structure

**Figure 15:**
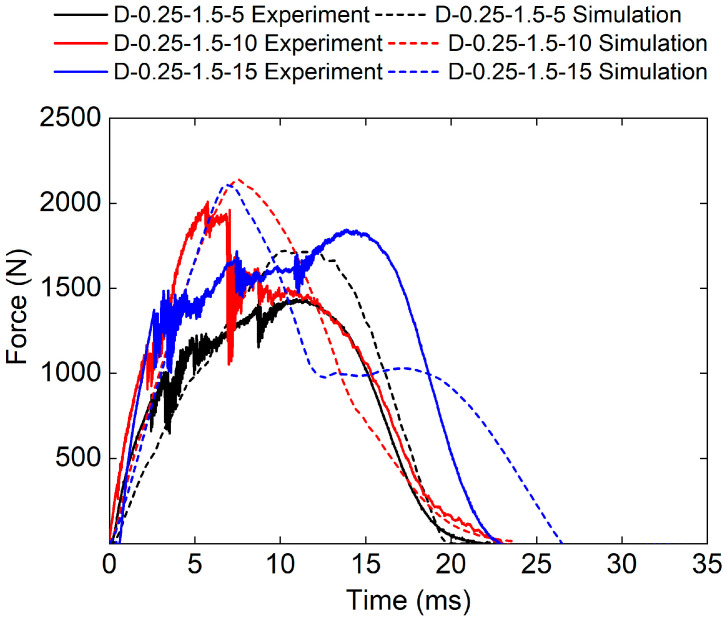
Experimental and simulation force–time curves of TPMS sandwich with different E.

Figure 15 is the force–time curve of the sandwich structure under different impact energies obtained from both the experimental and numerical methods. It can be seen that the force–time curve trends in the three sandwich structures are generally consistent. However, the simulated impact load is bigger than that of experimental results for D-0.25-1.5-15 sandwich structures. This is because the higher-impact energy leads to more damage to the panel and core, which cannot be captured by the maximum principal strain failure criterion used in the FE model.

The experimental peak loads of the D-0.25-1.5-5, D-0.25-1.5-10, and D-0.25-1.5-15 sandwich structures are 1442.35 N, 2010.4 N, and 1845.13 N, respectively. The simulated peak loads of the D-0.25-1.5-5, D-0.25-1.5-10, and D-0.25-1.5-15 sandwich structures are 1726.73 N, 2020.66 N, and 2111.97 N, respectively. The relative errors between experimental peak loads and simulated peak loads are 19.7%, 0.5%, and 14.5%, respectively.

**Figure 16:**
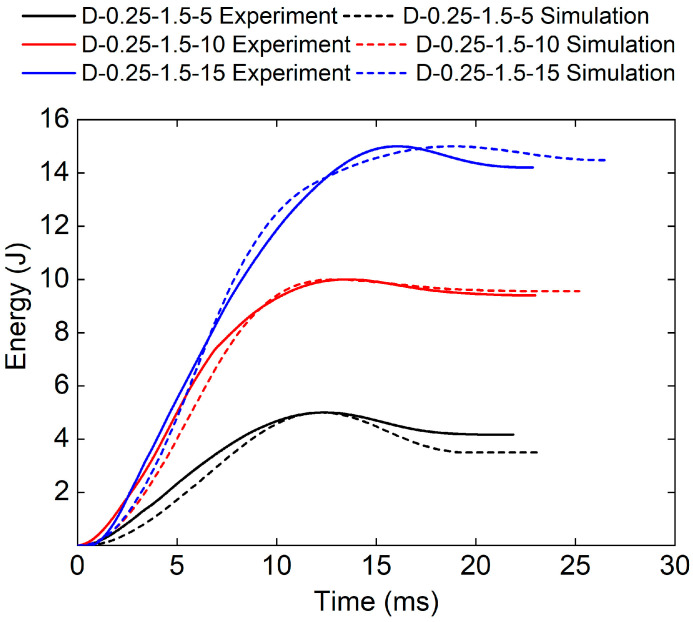
Comparison of energy–time curves between test and simulation of TPMS sandwich with different E.

Figure 16 and Table 6 show the experimental and numerical results of TPMS sandwich structures with different impact energies. The higher the impact energy, the bigger the proportion of absorbed energy (83.2% for D-0.25-1.5-5, and 94.7% for D-0.25-1.5-15). The reason may be that only a small part of the TPMS sandwich failed and fractured during impact leading to the absorbed energy is small.

**Figure 17:**
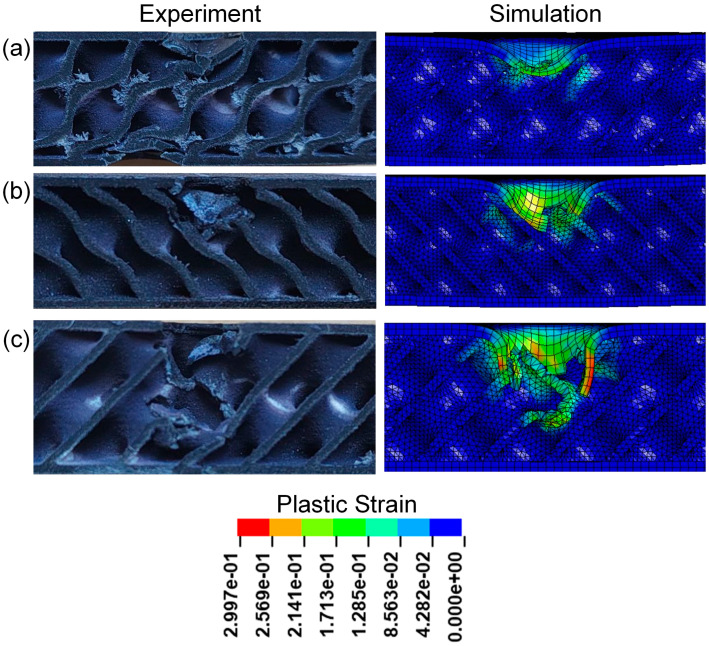
Comparison of the experimental and simulation impact damage of TPMS sandwich with different E: (**a**) D-0.25-1.5-5; (**b**) D-0.25-1.5-10; (**c**) D-0.25-1.5-15.

Figure 17 presents the cross-sectional view of sandwich structures with different impact energies after impact. Observing the core damage of the three sandwich structures under different impact energy, it is found that the panel of the sandwich structure is damaged and the core layer is broken. The core of the D-0.25-1.5-5 sandwich structure is slightly broken after the panel is destroyed, and both the panel and core of the TPMS sandwich structure with an impact energy of 10 J are destroyed. The damage of the impact area of the D-0.25-1.5-15 sandwich structure is the most serious, and the lower panel is slightly broken.

**Figure 18:**
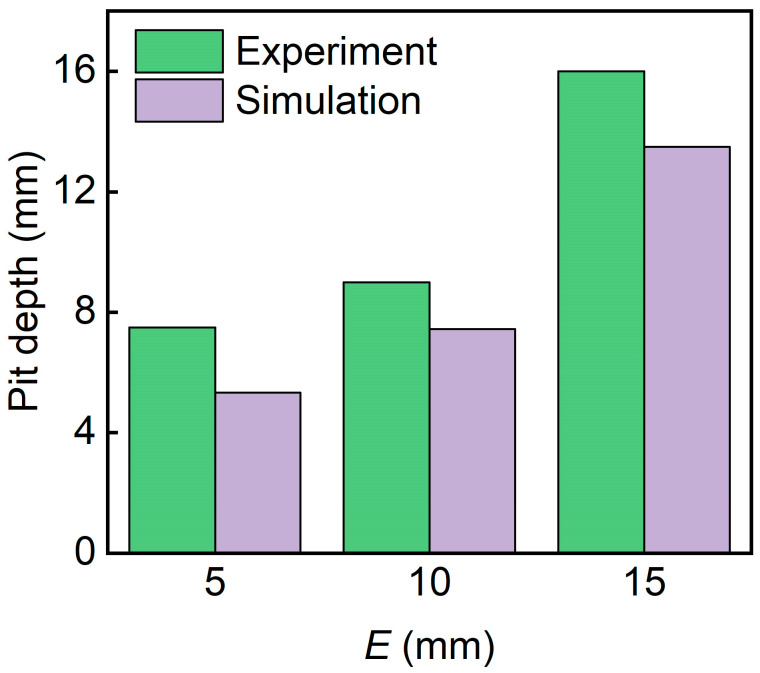
Experimental and simulation pit depths of TPMS sandwich with different E.

Figure 18 shows the pit depths of sandwich structures with different impact energies. It can be seen that pit depth increases with the increase in impact energy. The D-0.25-1.5-15 sandwich has the biggest pit depth. The simulated pit depths are in good agreement with the experimental pit depths.

#### 3.2.4. Influence of TPMS Core on Impact Performance of Sandwich Structures

**Figure 19:**
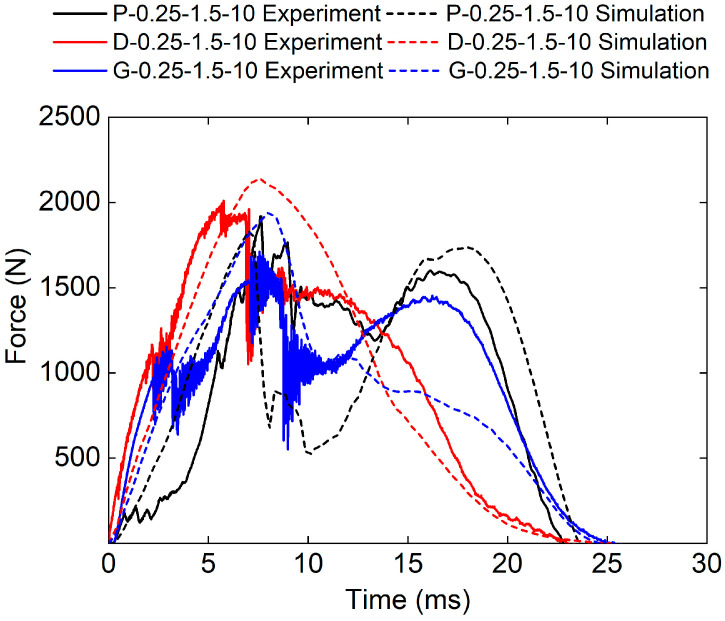
Experimental and simulation force–time curves of TPMS sandwich with different TPMS cores.

Figure 19 shows the force–time curves of different TPMS cores obtained from both the experimental and numerical methods. It can be seen that the experimental and numerical force–time curves exhibit similar trends. The D-0.25-1.0-10 sandwich structure has the highest impact stiffness.

The experimental peak loads of the P-0.25-1.0-10, G-0.25-1.0-10, and D-0.25-1.0-10 sandwich structures are 1918.17 N, 1711.57 N, and 2010.4 N, respectively. The simulated peak loads of the P-0.25-1.0-10, G-0.25-1.0-10, and D-0.25-1.0-10 sandwich structures are 1703.32 N, 1628.28 N, and 2020.66 N respectively. The relative errors between experimental peak loads and simulated peak loads are −11.2%, 0.5%, and 0.5%, respectively.

**Figure 20:**
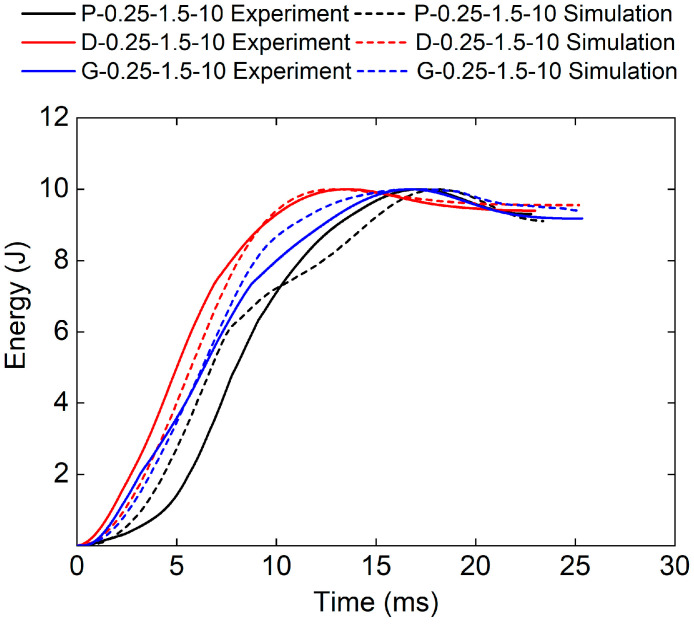
Comparison of energy–time curves between test and simulation of TPMS sandwich with different TPMS cores.

**Figure 21:**
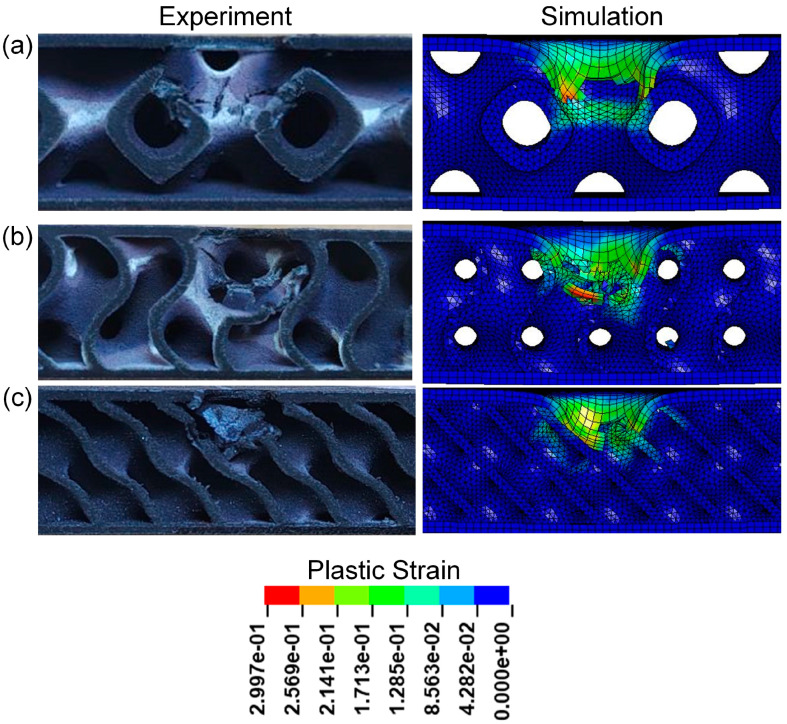
Comparison of the experimental and simulation impact damage of TPMS sandwich with different TPMS cores: (**a**) P-0.25-1.5-10; (**b**) G-0.25-1.5-10; (**c**) D-0.25-1.5-10.

Figure 20 and Table 7 show the experimental and numerical results of TPMS sandwich structures with different TPMS cores. It can be seen that the D-0.25-1.5-10 sandwich structure has the longest impact time and absorbed energy. Figure 21 shows the cross-sectional view of sandwich structures with different TPMS cores after impact. It can be seen that the D-TPMS core exhibits a more even distribution of the material and the failure and fracture of the D-TPMS core are more severe, which leads to more dissipation of energy.

It can be seen from Figure 21a that in the low-velocity impact experiment of the P-0.25-1.5-10 sandwich structure, the upper panel is damaged first, and then it comes into contact with the core layer through a space, in which the cells on the left side of the impact area are more severely damaged than those on the right side. It is obvious from Figure 21b,c that the impact zone of the G-0.25-1.5-10 and D-0.25-1.5-10 sandwich structure is seriously damaged, and the panel and core layer are fractured.

**Figure 22:**
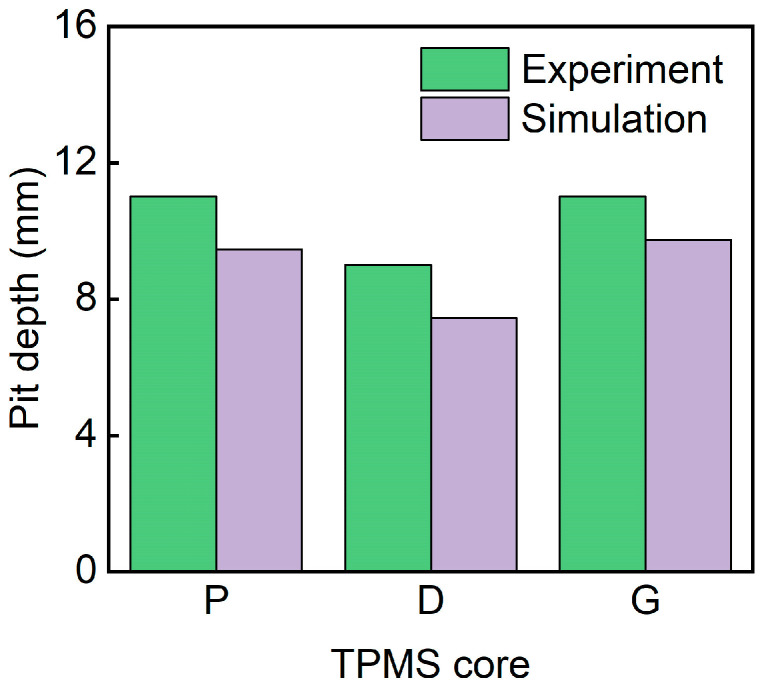
Comparison of the experimental and simulation pit depth of TPMS sandwich with different TPMS cores.

Figure 22 shows the pit depth of sandwich structures with different TPMS cores. The simulated pit depths for all three structures are in good agreement with the experimental results. The D-0.25-1.5-10 exhibits the smallest pit depth. This is because the D-TPMS core exhibits a more compact configuration, which may lead a uniform damage under impact loading.

## 4. Conclusions

The TPMS sandwich was proposed using the TPMS structure as the core, and their low-velocity impact responses were investigated using experimental and numerical methods. Parameter studies were conducted using FE analysis to study the effect of structural parameters (relative density, panel thickness, impact energy, and TPMS core) on the impact performances of the TPMS sandwich structures. The conclusions are as follows.

(1) The higher the relative density of the Diamond sandwich structure, the faster the time to reach the peak load and the greater the peak load. The greater the relative density, the stronger the impact resistance of the Diamond sandwich structure.

(2) The peak load of the Diamond sandwich structure increases with the increase in panel thickness, and the time to reach the peak load decreases with the increase in panel thickness. This shows that the greater the thickness of the panel, the stronger the impact resistance of the sandwich structure.

(3) The peak load of the Diamond sandwich structure increases with the increase in impact energy, and the greater the impact energy, the more severe the damage degree of the sandwich structure.

(4) The diamond sandwich structure has the largest peak load and better impact resistance compared with the P and G sandwich structures.

## Figures and Tables

**Table 1:** The geometric parameters and impact energy of the test samples.

Samples	*RD* (%)	$t_{f}$ (mm)	Impact Energy (J)	Numbers
D-0.25-1.5-5	0.25	1.5	5	1
D-0.25-1.5-10	0.25	1.5	10	2
D-0.25-1.5-15	0.25	1.5	15	1
D-0.25-1.0-10	0.25	1.0	10	1
D-0.25-2.0-10	0.25	2.0	10	1
D-0.2-1.5-10	0.2	1.5	10	1
D-0.3-1.5-10	0.3	1.5	10	1
G-0.25-1.5-10	0.25	1.5	10	1
P-0.25-1.5-10	0.25	1.5	10	1

**Table 2:** The process parameters for the MJF.

Ink Jet Speed (10,000 Drops/s)	Ambient Temperature (°C)	Powder Melting Point (°C)	Average Diameter of Powder (μm)	Bulk Density of Powder (g/cm^3^)
3000	20–30	187	60	0.425

**Table 3:** Material properties of PA12.

Density (g/cm^3^)	Elastic Modulus (MPa)	Poisson’s Ratio	Yield Strength (MPa)
1.01	1800	0.38	46

**Table 4:** Experimental and numerical results of TPMS sandwich with different RD.

Specimen	$\mathrm{Experimental}\;E_{ab}$ (J)	$\mathrm{Numerical}\;E_{ab}$ (J)
D-0.20-1.5-10	9.44	9.64
D-0.25-1.5-10	9.40	9.56
D-0.30-1.5-10	9.11	8.29

**Table 5:** Experimental and numerical results of TPMS sandwich with different $t_{f}$.

Specimen	$\mathrm{Experimental}\;E_{ab}$ (J)	$\mathrm{Numerical}\;E_{ab}$ (J)
D-0.25-1.0-10	9.21	9.70
D-0.25-1.5-10	9.40	9.56
D-0.35-2.0-10	8.65	7.29

**Table 6:** Experimental and numerical results of TPMS sandwich with different E.

Specimen	$\mathrm{Experimental}\;E_{ab}$ (J)	$\mathrm{Numerical}\;E_{ab}$ (J)
D-0.25-1.5-5	4.16	3.50
D-0.25-1.5-10	9.40	9.56
D-0.25-1.5-15	14.21	14.48

**Table 7:** Experimental and numerical results of TPMS sandwich with different TPMS cores.

Specimen	$\mathrm{Experimental}\;E_{ab}$ (J)	Numerical $E_{ab}$ (J)
P-0.25-1.5-10	9.30	9.11
D-0.25-1.5-10	9.40	9.56
G-0.25-1.5-10	9.17	9.38

## Data Availability

The original contributions presented in the study are included in the article, further inquiries can be directed to the corresponding author.
